# Comparative Transcriptome and Hormonal Analysis Reveals the Mechanisms of Salt Tolerance in Rice

**DOI:** 10.3390/ijms26146660

**Published:** 2025-07-11

**Authors:** Dingsha Jin, Yanchao Xu, Asif Iqbal, Yuqing Liu, Yage Zhang, Youzhen Lin, Liqiong Tang, Xinhua Wang, Junjie Wang, Mengshu Huang, Peng Xu, Xiaoning Wang

**Affiliations:** 1 Sanya Institute, Hainan Academy of Agricultural Sciences, Sanya 572000, China; jindingsha@163.com (D.J.); yuqing0304@sina.cn (Y.L.); zhangyage1992@163.com (Y.Z.); linyouzhen123@126.com (Y.L.); 18417278801@163.com (J.W.); woaixuexihms@foxmail.com (M.H.); 2 Institute of Food Crops, Hainan Academy of Agricultural Sciences, Haikou 571100, China; tangliqiong88@126.com (L.T.); wxh13876264156@163.com (X.W.); 3 National Nanfan Research Institute (Sanya), Chinese Academy of Agricultural Sciences, Sanya 572024, China; xuyanchao2016@163.com; 4 Department of Agriculture, Hazara University, Khyber Pakhtunkhwa, Mansehra 21120, Pakistan; asif173aup@gmail.com; 5 CAS Key Laboratory of Tropical Plant Resources and Sustainable Use, The Xishuangbanna Tropical Botanical Garden, Chinese Academy of Sciences, Menglun, Mengla 666303, China

**Keywords:** rice, seed germination, salt stress, transcriptome analysis, hormonal regulation, salt-tolerance mechanisms

## Abstract

Salt stress is a major constraint to seed germination and early seedling growth in rice, affecting crop establishment and productivity. To understand the mechanisms underlying salt tolerance, we investigated two rice varieties with contrasting responses as follows: salt-tolerant sea rice 86 (SR86) and salt-sensitive P559. Germination assays under increasing NaCl concentrations (50–300 mM) revealed that 100 mM NaCl induced clear phenotypic divergence. SR86 maintained bud growth and showed enhanced root elongation under moderate salinity, while P559 exhibited significant growth inhibition. Transcriptomic profiling of buds and roots under 100 mM NaCl identified over 3724 differentially expressed genes (DEGs), with SR86 showing greater transcriptional plasticity, particularly in roots. Gene ontology enrichment revealed tissue- and genotype-specific responses. Buds showed enrichment in photosynthesis-related and redox-regulating pathways, while roots emphasized ion transport, hormonal signaling, and oxidative stress regulation. SR86 specifically activated genes related to photosystem function, DNA repair, and transmembrane ion transport, while P559 showed activation of oxidative stress-related and abscisic acid (ABA)-regulated pathways. Hormonal profiling supported transcriptomic findings as follows: both varieties showed increased gibberellin 3 (GA3) and gibberellin 4 (GA4) levels under salt stress. SR86 showed elevated auxin (IAA) and reduced jasmonic acid (JA), whereas P559 maintained stable IAA and JA levels. Ethylene precursor and salicylic acid levels declined in both varieties. ABA levels rose slightly but not significantly. These findings suggest that SR86’s superior salt tolerance results from rapid growth, robust transcriptional reprogramming, and coordinated hormonal responses. This study offers key insights into early-stage salt stress adaptation and identifies molecular targets for improving stress resilience in rice.

## 1. Introduction

Plants are continually exposed to a variety of biotic and abiotic stresses throughout the year. Among these, soil salinity is the predominant abiotic stress significantly affecting seed germination, plant growth, and overall crop productivity worldwide [1,2]. Currently, approximately 20% of irrigated agricultural land across the globe is affected by soil salinity [3]. Rice (*Oryza sativa* L.) is a staple food for over half of the world’s population and is traditionally considered a salt-sensitive crop [4]. Nevertheless, rice possesses unique potential for saline–alkali soil reclamation, making it the preferred cereal crop for the productive use of marginal lands.

Soil salinity leads to multiple adverse effects on plants, including water deficit, osmotic stress, ion toxicity, reduced photosynthesis, oxidative stress, nutrient imbalances, all of which collectively inhibit normal plant growth development [5]. High salinity exacerbates oxidative stress and restricts root elongation [6]. Excess salinity (such as Na^+^) in the soil reduces the water potential around the root zone, thereby limiting water availability to the plant, and leading to osmotic stress [7]. Osmotic stress reduces cell turgor, disturbs water relations, and negatively affects water-use efficiency in plants [8,9]. It also triggers stomatal closure and degradation of photosynthetic pigments, ultimately decreasing photosynthetic efficiency [10,11].

Exposure to excessive NaCl leads to significant Na^+^ accumulation in the roots of *Arabidopsis* [12]. Sodium (Na^+^) and chloride (Cl^−^) ions absorbed from saline soils are transported through the xylem to shoots, eventually accumulating in the leaves where photosynthesis occurs [4,13]. Salt stress also triggers the rapid production of reactive oxygen species (ROS) in plants, such as hydrogen peroxide (H_2_O_2_), singlet oxygen (^1^O_2_), superoxide (·O_2_−), and hydroxyl radicals (OH), primarily due to metabolic disruptions [14]. These ROS can impose oxidative damage to vital macromolecules such as lipids, nucleic acids, proteins, and carbohydrates, resulting in redox imbalance and severe oxidative stress in plants [15]. Ultimately, oxidative stress impairs the structure and function of cellular organelles, further exacerbating the damage [5]. Collectively, these adverse effects hinder normal plant growth and development.

Seed germination is the most critical stage in the growth and development of crops, ultimately affecting both yield and quality of crops [16]. Successful germination depends heavily on favorable environmental conditions, which initiate plant growth [17]. Phytohormones such as abscisic acid (ABA) and gibberellins (GA), along with auxins, ethylene, and jasmonic and salicylic acid, interact through complex molecular pathways to regulate the rupture of the seed coat, enabling radicle emergence and nutrients mobilization from the endosperm to support early growth [17]. Among these, ABA and GA are the primary hormones controlling seed dormancy and germination in an antagonistic manner [18,19]. ABA and its signaling pathways inhibit germination by promoting dormancy, whereas GA promotes germination by counteracting ABA’s effects [18,19,20,21]. Salt stress results in hyperosmotic signal, triggering ABA accumulation, which initiates various adaptive responses in plants [22]. However, during seed germination, salt stress inhibits water absorption, damages root cell structure, disrupts enzymatic activity and disturbs hormonal balance, all of which negatively affect germination. Therefore, understanding salt tolerance mechanisms during seed germination is essential for sustaining rice production and developing salt tolerant cultivars.

Phytohormones play pivotal roles in mediating salt stress responses in rice. A previous study found that the ethylene signaling activators *OsEIN2/MHZ7* act as negative regulators in rice salt stress responses [23]. Compared to the wild-type, the mhz7/osein2-1 mutant exhibited significantly enhanced seedling survival after 5 days of salt treatment, whereas *OsEIN2/MHZ7* overexpression lines showed reduced survival [23]. In rice, overexpressing ABA-responsive factor *Rab16A*, confers enhanced salt tolerance and superior physiological performance in transgenic plants [24]. *OsJAZ9* fine tunes JA-responsive gene expression through an assembly of a transcriptional regulatory complex with OsNINJA and OsbHLH062, thereby contributing to salt stress tolerance in rice [25]. *RST1* encoded an auxin response factor (OsARF18). Loss of *RST1* function upregulated the expression of OsAS1 and improved nitrogen (N) utilization through promoting asparagine biosynthesis and avoiding excess ammonium (NH4^+^) accumulation, ultimately improving plant salt tolerance and yield [26].

The plant salt stress response represents a sophisticated regulatory program integrating osmotic adjustment, ionic homeostasis reconfiguration, ROS scavenging, and hormone metabolism, which collectively induces cascading transcriptomic, metabolic, and physiological alterations. With the development of transcriptomic technologies, researchers can find more regulatory genes [27,28,29,30]. Recent advances in metabolomics enable comprehensive detection of plant metabolites, providing a powerful tool for scientific research [31,32]. At present, multi-omics data integration, e.g., transcriptomics and metabolomics analysis, have provided effective resolution into the molecular and physiological foundations of abiotic stress adaptation [33,34,35].

Sea rice 86 (SR86) is a salt-tolerant rice cultivar that also possesses several beneficial traits, such as submergence and water logging tolerance, disease and pest resistance, and the ability to grow in infertile and marginal lands. Although previous studies have investigated the salt-tolerance mechanism of SR86, they remain incompletely understood. To further elucidate these mechanisms, this study employed RNA sequencing (RNA-seq) and hormone metabolome analysis on buds and roots of the salt-sensitive cultivar P559 and the salt-tolerant cultivar SR86 under both normal and salt stress conditions during seed germination. Comparative analysis of transcriptomic and hormonal profiles between SR86 and P559 revealed key adaptive responses to salt stress during the germination and provided a theoretical basis for the development of new salt-tolerant rice varieties.

## 2. Results

### 2.1. Phenotypic Response of Rice to Salt Stress

**Figure 1:**
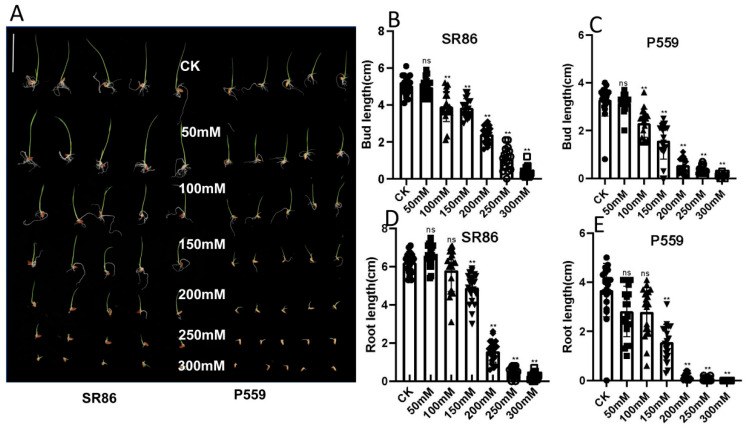
The influence of salt stress (4-day exposure) on seed germination. (**A**): Phenotype of germination of SR86 and P559 seeds under different concentrations of NaCl; (**B**): Bud length of SR86; (**C**): Bud length of P559; (**D**): Root length of SR86; (**E**): Root length of P559. Note: ●, ■, ▲, ▼, ◆, ○ and □ represent data under different concentrations of NaCl; ns and ** indicate no significant difference and significant difference (*p* < 0.01) compared to the control.

Seed germination under salt stress was evaluated in two rice varieties (salt-tolerant; SR86 and salt-sensitive; P559). Phenotypic evaluations of bud and root lengths were measured at 4- and 10-days post-treatment (dpt) under control (H_2_O) and six NaCl concentrations ranging from 50 to 300 mM. Both varieties exhibited significant reductions in bud and root elongation with increasing salt stress (Figure 1 and Figure S1, Table S1). At 4 dpt, 50 mM NaCl had no significant effect on bud or root length in either variety (Figure 1 and Table S1). However, exposure to 100 mM NaCl caused a marked reduction in bud length, while root length remained unaffected (Figure 1 and Table S1). By 10 dpt, varietal differences became evident as SR86 maintained bud growth, similar to controls under 100 mM NaCl, whereas P559 displayed significant bud inhibition. Additionally, SR86 exhibited enhanced root elongation under 50–100 mM NaCl, a response that was absent in P559 (Figure S1). These findings highlight SR86’s superior salt tolerance, characterized by sustained bud development and compensatory root elongation under moderate salinity. In contrast, P559 demonstrated limited phenotypic plasticity. Based on these contrasting responses at 100 mM NaCl, this concentration was selected for subsequent RNA-sequencing experiments to investigate the molecular mechanisms of salt tolerance.

**Figure 2:**
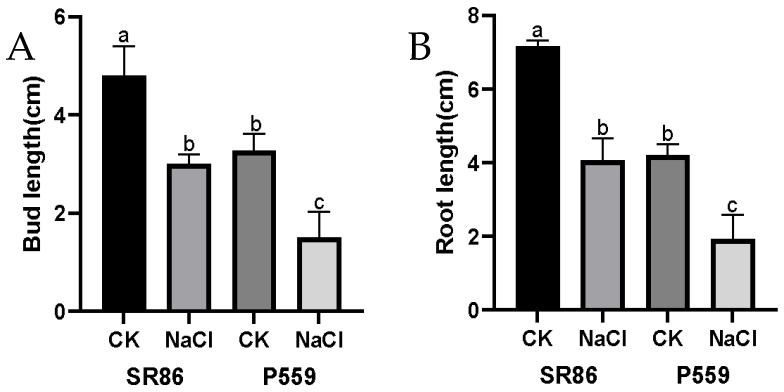
Effects of salt stress on bud and root length. (**A**) Bud length of SR86 and P559; (**B**) Root length of SR86 and P559 under control and 100 mmol/L NaCl. Different letters in the data columns indicate significant differences (*p* < 0.05) according to Duncan’s test.

To investigate the basis of SR86’s superior germination vigor and salt tolerance, comparative analyses of bud and root elongation were performed under 100 mM NaCl. Under control (H_2_O) conditions, SR86 exhibited significantly longer buds and roots than P559, indicating an inherent growth advantage in the absence of stress (Figure 2A,B and Table S2). Exposure to 100 mM NaCl suppressed both traits in both varieties, though SR86 maintained relatively greater elongation capacity compared to P559 (Figure 2A,B and Table S2). These results suggest that SR86 enhanced salt tolerance may be partially attributed to its accelerated growth dynamics during germination, enabling faster seedling establishment under stress. The combination of constitutive growth vigor under non-stress conditions and maintained elongation under salinity supports the hypothesis that rapid tissue expansion during early development contributes to SR86’s adaptive response to stress.

### 2.2. Comparative Transcriptome Analysis of Two Rice Genotypes with Contrasting Salt Tolerance

This study leverages the observed morphological differences between SR86 and P559 under 100 mM NaCl, a concentration that elicited clear differential growth responses. To elucidate the molecular basis of SR86’s enhanced salt tolerance, transcriptomic comparisons were conducted in actively growing tissues (buds and roots) of both genotypes during early seedling establishment. RNA sequencing was performed on samples collected under 100 mM NaCl treatment, targeting salt-responsive gene expression in both tissues. The sequencing yielded high-quality data with 1.82 Gb clean reads across all samples (Table S3). Mapping to reference the rice genome demonstrated high alignment efficiency, with uniquely mapped reads ranging from 83.53 to 94.83%, and properly mapped reads from 81.28 to 91.97% (Table S3), confirming the reliability of the dataset for downstream transcriptomic analyses.

**Figure 3:**
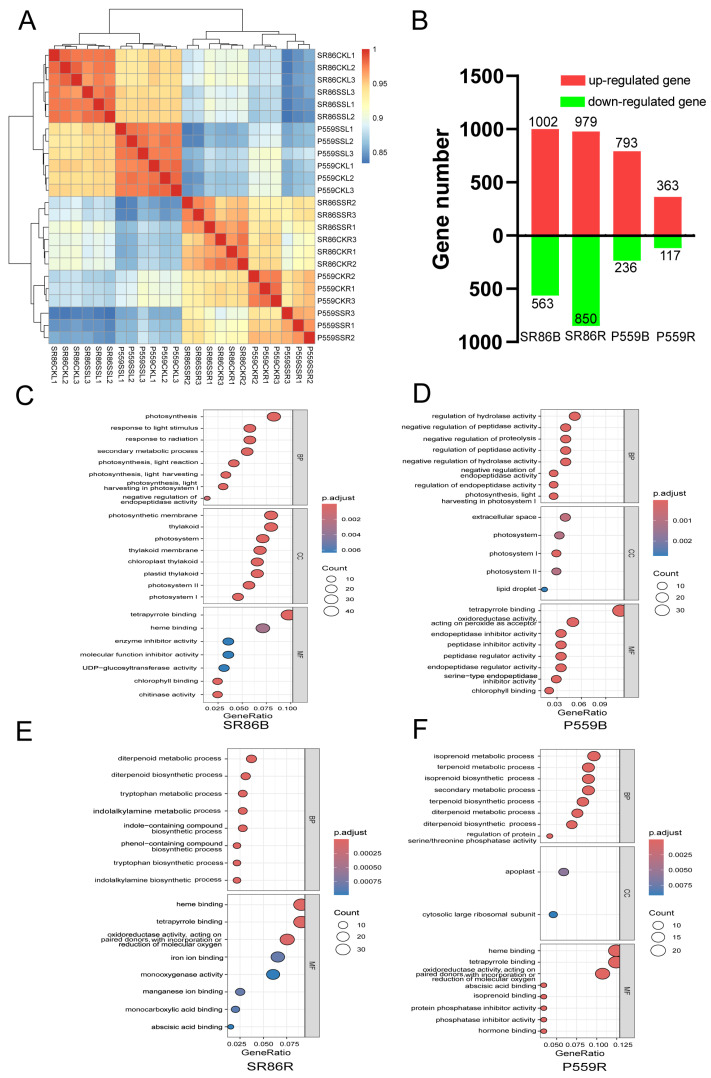
The number of differentially expressed genes (DEGs) and the GO analysis of DEGs at germination. (**A**): Pearson correlation analysis of DEGs; (**B**): The number of DEGs between four groups, i.e., SR86CKB vs. SR86SSB (SR86B), SR86CKR vs. SR86SSR (SR86R), P55986CKB vs. P559SSB (P559B), P559CKR vs. P559SSR (P559R); (**C**): GO enrichment of DEGs at SR86B group; (**D**): GO enrichment of DEGs at P559B group; (**E**): GO enrichment of DEGs at SR86R group; (**F**): GO enrichment of DEGs at P559R group.

To investigate the transcriptional divergence between SR86 and P559 rice varieties under salt stress, we performed comparative analyses of gene expression patterns across tissues using principal component analysis (PCA, Figure S2) and Pearson correlation coefficient analysis (Figure 3A). The PCA results demonstrated clear stratification of samples based on both tissue type (bud vs. root) and genetic background (SR86 vs. P559). Notably, buds exhibited marked transcriptional differences compared to roots, and the two rice varieties were clearly separated in expression space. These spatial and genotypic distinctions were further supported by the Pearson correlation analysis, which revealed stronger intra-tissue than inter-tissue correlations. Collectively, these findings highlight the presence of tissue-specific and genotype-dependent regulatory mechanisms in responses to salt stress, suggesting that rice tissues and genetic backgrounds may employ differential molecular adaptation strategies under saline conditions.

RNA sequencing revealed substantial differences in the transcriptional responses of SR86 and P559 rice varieties to salt stress, identifying approximately 3724 differentially expressed genes (DEGs). SR86 exhibited a more pronounced transcriptional reprogramming, particularly in its root tissues. Specifically, SR86 buds contained 1565 DEGs (563 downregulated, 1002 upregulated), while roots showed 1829 DEGs (850 downregulated, 979 upregulated). In contrast, P559 displayed more limited transcriptional changes, with 1029 DEGs in buds (236 downregulated, 793 upregulated) and only 480 DEGs in roots (117 downregulated, 363 upregulated). The substantially higher number of DEGs in SR86, particularly in roots, indicates that it engages a more dynamic and complex gene regulatory network in response to salt stress. This enhanced transcriptional plasticity likely contributes to SR86’s superior adaptability to saline environments.

**Figure 4:**
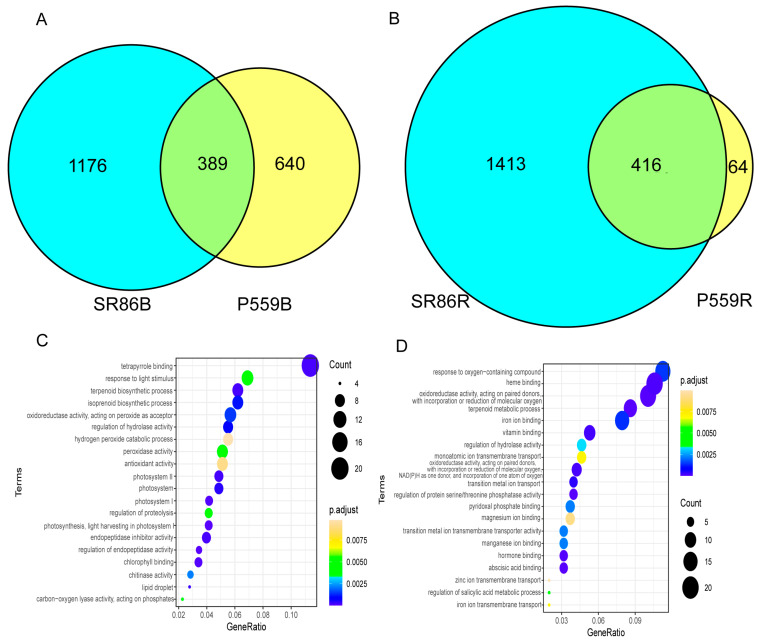
Venn diagrams of DEGs between two groups and GO enrichment analysis of DEGs. (**A**): Venn diagram indicating the numbers of common and specific DEGs in buds between SR86 and P559; (**B**): Venn diagram indicating the numbers of common and specific DEGs in rootss between SR86 and P559; (**C**): GO enrichment analysis of comment DEGs in buds between SR86 and P559; (**D**) GO enrichment analysis of comment DEGs in roots between SR86 and P559.

To explore specific pathways enriched by gene ontology (GO) analysis, GO terms were categorized into biological properties (BP), cellular component (CC), and molecular function (MF), using a significance threshold of q-value < 0.01. In the SR86 bud comparison (SR86B), a total of 19 BP, 17 CC, and 5 MF GO terms were significantly enriched (Table S4). In contrast, the P559 bud comparison (P559B) revealed enrichment of 25 BP, 4 CC, and 18 MF GO terms (Table S5). Among the BP terms, pathways associated with peptidase activity, photosynthesis, and terpenoid metabolic processes were predominant in both comparisons. In the CC category, significant terms included photosystem, photosynthetic membrane, thylakoid membrane, and organelle outer membrane. For MF, key enriched terms included tetrapyrrole binding, chlorophyll binding, chitinase activity, and oxidoreductase activity, all of which play an important role in the plant’s response to salt stress (Figure 3C,D, Tables S2 and S3). GO enrichment analysis highlighted both shared and distinct biological mechanisms in the salt stress responses of SR86 and P559 buds. Both varieties exhibited convergent activation of photosynthesis-related pathways such as photosynthesis, light harvesting in photosystem I, chlorophyll binding, tetrapyrrole binding, and endopeptidase regulator activity (Figure 4C,D), highlighting conserved roles of photosynthetic adjustment and protein homeostasis salt tolerance.

In SR86 root comparison (SR86R), 34 GO terms were classified under BP and 18 GO terms were classified as MF (Table S6). In P559 root comparison (P559R), 29 GO terms were classified as BP, and 29 GO terms were classified as MF (Table S7). The GO terms, enriched in SR86 and P559 roots in response to salt stress, included processes such as terpenoid metabolic process, isoprenoid metabolic process, regulation of protein serine/threonine phosphatase activity, transition metal ion transport, and regulation of salicylic acid metabolic process. Additionally, terms related to MFs, such as tetrapyrrole binding, oxidoreductase activity, acting on paired donors, with incorporation or reduction in molecular oxygen, oxidoreductase activity, acting on paired donors, with incorporation or reduction in molecular oxygen, NAD(P)H as one donor, incorporation of one atom of oxygen, vitamin binding, iron ion binding, monooxygenase activity, manganese ion binding, and transition metal ion transmembrane transporter activity. These shared GO terms underscore conserved mechanisms for maintaining cellular ion homeostasis and metabolic flexibility under salt stress (Figure 3E,F, Tables S6 and S7).

In this study, we identified 389 common DEGs in buds and 416 common DEGs in roots of SR86 and P559 under salt stress during germination (Figure 4). GO enrichment of these shared DEGs revealed distinct tissue-specific functional specialization. In buds, 38 significantly enriched GO terms were primarily linked to photosystem function (e.g., light harvesting, chlorophyll binding), proteostasis regulation (endopeptidase activity), and redox homeostasis (oxidoreductase activity) (Figure 4C, Table S8). In contrast, roots exhibited broader functional adaptation, with 56 enriched GO terms related to terpenoid/isoprenoid metabolism, metal ion transport regulation, oxidoreductase activity, phosphatase activity modulation, and hormone-related processes (e.g., salicylic acid and abscisic acid pathways) (Figure 4D, Table S9).

Despite these tissue-specific enrichments, oxidation-reduction processes were commonly enriched in both buds and roots, underscoring the critical role of ROS scavenging in salt tolerance. Photosystem-related genes were highly represented in buds, suggesting that adjustment to photosynthetic processes are vital for protecting emerging aerial tissues under salinity. Meanwhile, roots activated specialized networks involved in ion transmembrane transport (e.g., zinc, cadmium, and inorganic anions) and hormone signaling (ABA/SA pathways), reflecting their role in regulating ionic and hormonal balance. This tissue-divergent enrichment pattern highlights a strategic resource allocation during early seedling development under salt stress as follows: above-ground plant parts prioritize energy metabolism and oxidative defense, while roots focus on ion exclusion, oxidative defense, and hormonal coordination. Together, these responses reflect a dual strategy that balances immediate stress mitigation with systemic regulation during germination.

### 2.3. Changes in Endogenous Hormones Exposed to Salt Stress

**Figure 5:**
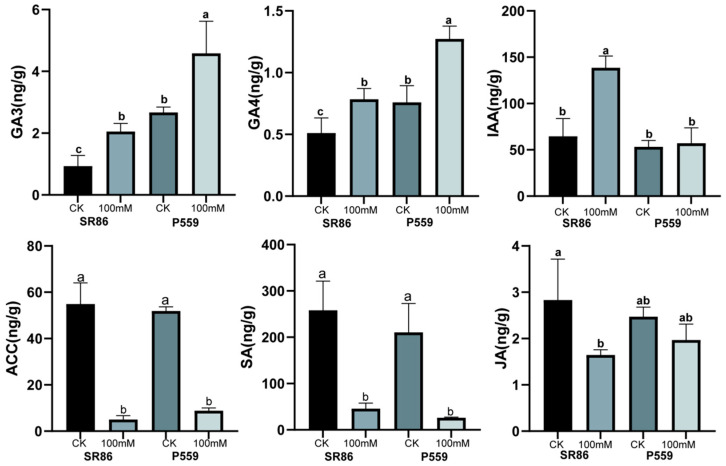
The effect of the endogenous hormone under salt stress. Note: Different letters in the data columns indicate significant differences (*p* < 0.05) according to Duncan’s test.

Transcriptome data analysis revealed that pathways related to photosynthesis, ion transport, antioxidant activity, and hormone metabolism may play important roles in the response to salt stress during seed germination. Previous studies have shown that ABA and GA hormones are critical regulators of seed germination. Therefore, this study investigated the changes in hormones during seed germination under salt stress conditions. Following a 4-day salt stress treatment, GA levels (GA_3_ and GA_4_) significantly increased in the seedlings of both rice varieties. Auxin (IAA) concentrations increased significantly in SR886 under salt stress while remaining unchanged in P559 (Figure 5 and Table S10). In contrast, levels of ethylene precursor ACC and salicylic acid (SA) significantly decreased in both rice varieties. Jasmonic acid (JA) levels decreased in SR86, while no significant change was observed in P559 under salt stress conditions (Table S10). The above results indicate hormonal regulation strategies and synergistic interactions among different phytohormones in response to salt stress. Although ABA levels increased in both varieties under salt stress, the changes were not statistically significant compared to the control (Figure S3).

## 3. Discussion

Salt stress has a predominant impact on seed germination, plant growth and overall crop productivity [1,2]. Soil salinity leads to water deficit, osmotic and ion stress, reduced photosynthesis, oxidative stress, and nutrient imbalance in plants [5]. In this study, rice seed germination was notably suppressed under salt stress, with the growth of P559 (salt-sensitive genotype) being more severely inhibited than SR86 (salt-tolerant genotype). This study aimed to investigate the physiological and molecular mechanisms underlying the rice response to salt stress.

### 3.1. Changes in Gene Transcription of Rice Under Salt Stress

Transcriptome analyses were conducted to explore salt-responsive metabolic pathways in rice. The highest number of DEGs was detected in SR86 with 1829 DEGs in the root tissues and 1565 DEGs in bud tissues. In contrast, P559 displayed fewer transcriptional changes in buds (1029 DEGs) and roots (480 DEGs). This suggests greater gene activity and transcriptional plasticity in the salt-tolerant genotype (SR86) exhibiting rapid activation of downstream protective mechanisms. This aligns with previous studies where the salt-tolerant rice genotype showed stronger transcriptomic reprogramming than sensitive ones [27]. In both genotypes, more genes were up-regulated than down-regulated, suggesting a proactive gene expression strategy to regulate growth and stress response under saline conditions [36]. These results indicate that rice is mobilizing gene expression to adapt to long-term salt stress, thereby regulating plant growth and development under salt stress conditions.

GO enrichment analysis highlighted both shared and distinct biological mechanisms underlying the salt stress responses in the buds and roots of SR86 and P559. In the buds of both varieties, there was a convergent activation of photosynthesis-related pathways (e.g., photosynthesis, light harvesting in photosystem I, photosynthesis), chlorophyll binding, tetrapyrrole binding, and endopeptidase regulator activity (Figure 4C,D). These results highlight the conserved roles of photosynthetic adjustment and protein homeostasis in the early response of rice buds to salt stress.

In the roots, enriched GO terms included terpenoid and isoprenoid metabolism, metal ion transport, regulation of salicylic acid metabolism, tetrapyrrole binding, and oxidoreductase activity. These findings reflect both ion transport and oxidative stress regulation mechanism, where plants activate metal ion transporters to mitigate Na^+^ toxicity and mobilize antioxidants to scavenge excessive ROS generated under salt stress. The regulation of protein serine/threonine phosphatase activity and tetrapyrrole binding suggests involvement in post-translational signaling cascades and chlorophyll metabolism, which are linked to redox and energy homeostasis. Additionally, shared terms included vitamin binding, iron ion binding, monooxygenase activity, manganese ion binding, transition metal ion transmembrane transporter activity, underscoring conserved strategies for maintaining cellular ion balance and metabolic flexibility under salinity.

Despite these shared responses, genotypes-specific enrichment profiles highlighted divergent adaptive priorities. In P559 roots, salt stress uniquely activated hormone-mediated regulation through abscisic acid (ABA) binding and carbohydrate metabolism via hydrolase activity, acting on glycosyl bonds, and regulation of phosphatase activity. These changes reflect a generalized stress signaling response but may be insufficient for effective adaptation. In contrast, SR86 roots exhibited enrichment of genes involved in inorganic ion and salt transmembrane transport, including zinc, cadmium, and monovalent ions. This demonstrates a specialized hyperosmotic signaling and ion transport response that enables SR86 to maintain osmotic balance and prevent toxic ion accumulation.

### 3.2. Buds and Roots Synergistically Regulate Antioxidant Pathways to Cope with Salt Stress

GO enrichment analysis showed that the distribution of enriched DEGs under salt stress was roughly similar between bud and root tissues of both rice cultivars. In bud tissues, commonly enriched GO terms were primarily linked to photosystem function, endopeptidase activity, and redox homeostasis (oxidoreductase activity), indicating these pathways play a vital role in the response of rice plants to salt stress (Figure 4C). Similarly, Wang et al. reported that in rice leaves, DEGs under salt stress were mainly related to peroxidase activity, magnesium ion binding, protein serine/threonine phosphatase activity, and cysteine-type endopeptidase activity [37]. Salt stress is known to cause imbalance, reduce photosynthesis, oxidative stress, and nutrient imbalance in plants [5]. Exposure to high salinity stress triggers excessive reactive oxygen species (ROS) accumulation in plant cells, which impairs critical physiological processes including photosynthesis and nutrient assimilation, ultimately leading to oxidative damage and cell death [15]. The mitochondrial glutathione peroxidase gene *OsGPX3* enhances salt tolerance in rice by reducing ROS accumulation [38]. Peroxisome-related and catalase-encoding genes potentiate salinity tolerance in rice through coordinated upregulation of key antioxidant enzymes (SOD, POD, CAT), which effectively scavenge ROS and attenuate oxidative membrane damage via the suppression of lipid peroxidation.

GO analysis further showed that roots exhibited broader functional adaptation, with more enriched pathways related to terpenoid/isoprenoid metabolism, metal ion transport regulation, oxidoreductase activity, phosphatase activity modulation, and plant hormone-related signaling and metabolism (e.g., salicylic acid and abscisic acid pathways). The co-enrichment of redox-related terms in both buds and roots highlights the central role of antioxidant regulation in rice’s salt stress response. These findings highlight shared anti-oxidative biological mechanisms between SR86 and P559 across tissues and suggest a coordinated regulatory network underlying varietal adaptation to salinity. This insight provides a molecular basis for breeding strategies focused on enhancing salinity tolerance through targeted improvement of anti-oxidative pathway.

### 3.3. Regulation of Hormone Metabolic Pathway Exposed to Salt Stress

During seed germination, salt stress inhibits water absorption, damages root cell structure, alters enzyme activity, and disrupts hormonal metabolism. Previous studies have demonstrated that ABA and GA, along with auxins, ethylene, and salicylic acid, regulate seed germination through interconnected molecular pathways [17]. Among these, ABA and GA play primary and antagonistic roles in regulating seed dormancy and germination [18,19]. In this study, GA_3_ and GA_4_ significantly increased in both rice varieties under salt stress during the germination stage (Figure 5). Typically, plants under salt stress enter a “growth arrest” state; however, GA accumulation could alleviate this response by promoting seed germination and resuming early growth. As key hormone promoting seed germination and seedling growth, GA counteracts salt-induced suppression of seed imbibition and radicle emergence. Elevated GA levels likely facilitate the embryo, overcoming salt-imposed physical barriers by activating hydrolytic enzymes, thereby accelerating the endosperm reserves to support early seedling development under salinity. This suggests that gibberellin signaling counteracts salt-induced suppression of early growth. Although ABA levels also increased under salt stress in both varieties, these changes were not statistically significant compared to the control condition.

Under salt stress, auxin (IAA) concentrations increased significantly in SR86, while remaining unchanged in P559 (Figure 5). This is consistent with the observed phenotype of SR86, which maintains relatively rapid growth under salt stress in this study. The enhanced salt tolerance observed in SR86 may be closely associated with its ability to maintain higher endogenous auxin levels. The elevated IAA levels in SR86 may enhance salt stress adaptation through two key mechanisms as follows: (1) By promoting lateral root development and root hair formation, IAA enhances lateral root development and root hair formation, which increases the root surface area and improves water and nutrient uptake, thereby mitigating osmotic imbalance caused by salinity. (2) IAA may interact with genes associated with transmembrane ion transport, such as Na^+^/H^+^ antiporters or HKT transporters to maintain cellular ion balance. This coordination facilitates the sequestration or exclusion of excess Na^+^, contributing to cellular stability under saline conditions.

In both rice varieties, the contents of ethylene precursor ACC and salicylic acid (SA) significantly decreased under salt stress. Ethylene has been shown to negatively regulate salt tolerance, as elevated ACC levels are associated with reduced salt tolerance in Arabidopsis [39,40]. In this study, the reduction in ACC levels may result from the suppression of ACS or activation of ACO, allowing plants to minimize ethylene overaccumulation and its associated growth inhibition. This suggests an adaptive response to salt stress by modulating ethylene biosynthesis. Furthermore, evidence suggests crosstalk between ethylene and ABA signaling pathways under salt stress [39]. In the case of SA, its role appears dose dependent. While low concentrations enhance the antioxidant defense mechanism, high concentrations can lead to excessive ROS accumulation [41,42]. Since salt stress inherently causes ROS accumulation [14], the observed decrease in SA levels may serve as a protective mechanism to prevent ROS overaccumulation, which mediates cellular damage, particularly in membranes and organelles. Together, the findings of this study illustrate that rice plants activate multiple integrated pathways under salt stress. The tolerant genotype SR86 demonstrates coordinated activation of hyperosmotic signaling, ion transport, oxidative stress regulation, hormone signaling, and gene activity, allowing it to maintain growth, redox balance, and ion homeostasis more effectively than the sensitive P559. These mechanisms provide critical targets for improving salt tolerance through molecular breeding and genetic engineering approaches.

## 4. Materials and Methods

### 4.1. Plant Materials and Growth Conditions

In this study, we used two rice varieties—*indica* landrace SR86 (salt-tolerant cultivation) and P559 (salt-sensitive rice). Healthy seeds were selected and surface sterilized by soaking them in 20% sodium hypochlorite solution for 20 min, followed by rinsing them three times with distilled water. The seeds were then incubated at 37 °C and soaked for another 48 h until radicle emergence (indicated by white protrusions). Twenty seeds from each variety were transferred in 9 cm Petri dishes containing 25 mL of either distilled water (control, CK) or salt solutions at concentrations of 50, 100, 150, 200, 250, and 300 mM Sodium chloride (NaCl). Solutions of 50, 100, 150, 200, 250, and 300 mmol/L NaCl (≥99.8%, Catalog No. 10019318, GR grade, Sinopharm Chemical Reagent Co., Ltd, Shanghai China) were prepared by dissolving 2.922, 5.844, 8.766, 11.688, 14.610, and 17.532 g of guaranteed-grade NaCl (≥99.8%, Sinopharm, Cat. No. 10019318) in distilled water to a final volume of 1 L. The procedure for preparing different concentration NaCl solution is as follows: (a) Accurately weigh NaCl in a beaker; (b) Add 800 mL distilled water; (c) Stir magnetically (500 rpm, 30 min) until fully dissolved; (d) Transfer to a 1 L volumetric flask, rinsing the beaker 3 times (combine rinsates into the flask); (e) Bring to volume at the calibration mark and homogenize.

The seeds were cultivated in a growth chamber set at 28 °C/24 °C (day/night), with 75% relative humidity and an 18 h/6 h light/dark cycle (light intensity ~ 16,000 LUX) for 10 days. At 4- and 10-days post-salt stress exposure, root and bud lengths were quantified with a ruler (precision: 0.1 cm), using three biological replicates. Whole-plant phenotypes were photographically documented 4 days after exposure to salt stress using a high-resolution camera. For transcriptome and hormone analysis, the buds and roots of SR86 and P559 were harvested after 4 days of treatment under 0 mM and 100 mM NaCl. All samples (24 samples) were immediately frozen in liquid nitrogen and stored at −80 °C. Each treatment was performed using three biological replicates.

### 4.2. Measurement of Hormones

The plants of SR86 and P559 were collected for hormone measurement after 4 days of treatment with 0 mM and 100 mM NaCl for hormone quantification. The levels of GA, ABA, IAA, ACC, JA, and SA were measured using liquid chromatography (LC-MS), following the protocols described by Balcke [43,44,45]. The samples were extracted in 1 mL of ice-cold 50% acetonitrile (ACN, vol/vol), sonicated for 3 min and subsequently incubated at 4 °C for 30 min. After centrifugation (10 min, 12,000 rpm, 4 °C), the supernatant was transferred to microtubes. The supernatant was purified using RP-SPE cartridges pre-conditioned with 1 mL MeOH and 1 mL ddH_2_O_2_, followed by equilibration with 50% ACN. After loading samples, the cartridge were rinsed with 1 mL of 30% ACN, and the eluted fraction was collected. The purified extracts were then evaporated to dryness under a gentle stream of nitrogen gas and stored at −20 °C until analysis. For LC-MS analysis, the dried samples were dissolved in 200 uL of 30% ACN and hormone concentrations were determined.

### 4.3. RNA Sequencing and Analysis

The SR86 and P559 seedlings that were exposed to 0 mM and 100 mM NaCl for 4 days were named as follows: SR86-CK-Root (SR86CKR), SR86-NaCl-Root (SR86SSR), SR86-CK-Bud (SR86CKB), SR86-NaCl-Bud (SR86SSB), P559-CK-Root (P559CKR), P559-NaCl-Root (P559SSR), P559-CK-Bud (P559CKB), and P559-NaCl-Bud (P559SSB), respectively. A total of 24 samples were used for RNA extraction using Trizol reagent (Invitrogen crop, Carlsbad, CA, USA), following the manufacturer’s instructions. The total RNA concentration and purity were determined using Bioanalyzer 2100 and RNA 1000 Nano LabChip Kit (Agilent, CA, USA) with RNA integrity number (RIN) > 7.0. The cDNA sequencing libraries were constructed using a NEBNext Ultra RNA Library Prep Kit (New England Biolabs (Beijing) LTD., Beijing, China)following the manufacturer’s protocol. Unique index codes were added to each library to enable sample identification during sequencing. All libraries were sequenced using 150 bp paired ends reads on the DNBSEQ-T7 platform by Bgi Genomics Co., Ltd. The data discussed in this publication have been deposited in NCBI SRA as PRJNA1267251 (https://www.ncbi.nlm.nih.gov/sra/PRJNA, accessed on 23 May 2025).

### 4.4. Transcriptome Data Analysis

Clean reads were obtained by removing low quality reads (reads containing adapter, primer and nucleotide with q quality score < 20 and poly-N) from the raw data. The filtered clean reads of every sample were aligned to the *Oryza sativa* japonica Nipponbare reference genome (https://rapdb.dna.affrc.go.jp/, accessed on 16 July 2024) using HISAT2. Transcript abundance was calculated using the fragments per kilobase of exon per million mapped fragments (FPKM) method with FeatureCounts. The DEGs analysis between control and treatment were identified using the DESeq2 package in R, with a threshold of |log2fold change|≥1 and false discovery rate (FDR) < 0.05. Gene Ontology (GO) enrichment analysis was performed using clusterProfiler. GO enrichment analysis was performed using the clusterProfiler in R. GO annotations were obtained from the ENSEMBL database (dataset: osativa_er_gene). Enrichment significance was calculated using the hypergeometric test. To control for multiple testing, the Benjamini–Hochberg procedure was applied to calculate False Discovery Rate (FDR) adjusted *p*-values. GO terms with FDR < 0.01 were considered statistically significantly enriched. Enrichment plots were generated using clusterProfiler and ggplot2. The following pairwise comparisons were conducted and referred to as follows: SR86-CK-Root vs. SR86-NaCl-Root, SR86-CK-Bud vs. SR86-NaCl-Bud, P559-CK-Root vs. P559-NaCl-Root, and P559-CK-Bud vs. P559-NaCl-Bud are referenced as SR86-R, SR86-B, P559-R, and P559-B, respectively.

### 4.5. Statistical Analysis

Data processing was performed using Microsoft Excel. Bar graphs were generated using Graphpad Prism 9.0.0, while Venn diagrams, scatter plots, and heatmaps were created using R software(R version 4.4.3).

## 5. Conclusions

This study demonstrates that the salt-tolerant rice variety SR86 exhibits superior germination and seedling growth under salt stress compared to the sensitive variety P559. SR86 maintains bud and root elongation at moderate salinity (100 mM NaCl), supported by dynamic transcriptional changes and tissue-specific activation of pathways related to photosynthesis, ion transport, antioxidant defense, and hormonal signaling. Hormonal analysis revealed genotype-specific regulation, with SR86 showing increased gibberellin and auxin levels under salt stress, while ethylene precursor, salicylic acid, and jasmonic acid levels decreased in both varieties. These hormonal adjustments likely contribute to SR86’s enhanced stress resilience. Overall, SR86’s improved salt tolerance arises from coordinated early growth vigor, complex gene regulatory networks, and fine-tuned hormonal responses. These insights advance our understanding of salt tolerance mechanisms during rice seed germination and offer valuable targets for breeding salt-resistant rice cultivars. However, this study identified several gene regulatory networks governing salt tolerance responses but did not detect major-effect salt tolerance genes. In future work, we will integrate genetic population mapping and genome-wide association studies (GWAS) of natural populations to screen candidate salt tolerance genes, with the goal of identifying major-effect genes. This will provide a more comprehensive molecular theoretical basis and breeding information for developing salt-tolerant rice varieties.

## Data Availability

All data analyzed during this study are included in this article and its additional files.

## Supplementary Materials

The following supporting information can be downloaded at: https://www.mdpi.com/article/10.3390/ijms26146660/s1.
