# Supplementary material for: Comparative Transcriptome and Hormonal Analysis Reveals the Mechanisms of Salt Tolerance in Rice
Source: Int J Mol Sci. 2025 Jul 11;26(14):6660. doi: 10.3390/ijms26146660 (PMC12295843; doi:10.3390/ijms26146660)
Supplement: Supplementary file 1 [file ijms-26-06660-s001.zip › ijms-3694761-supplementary.pdf]

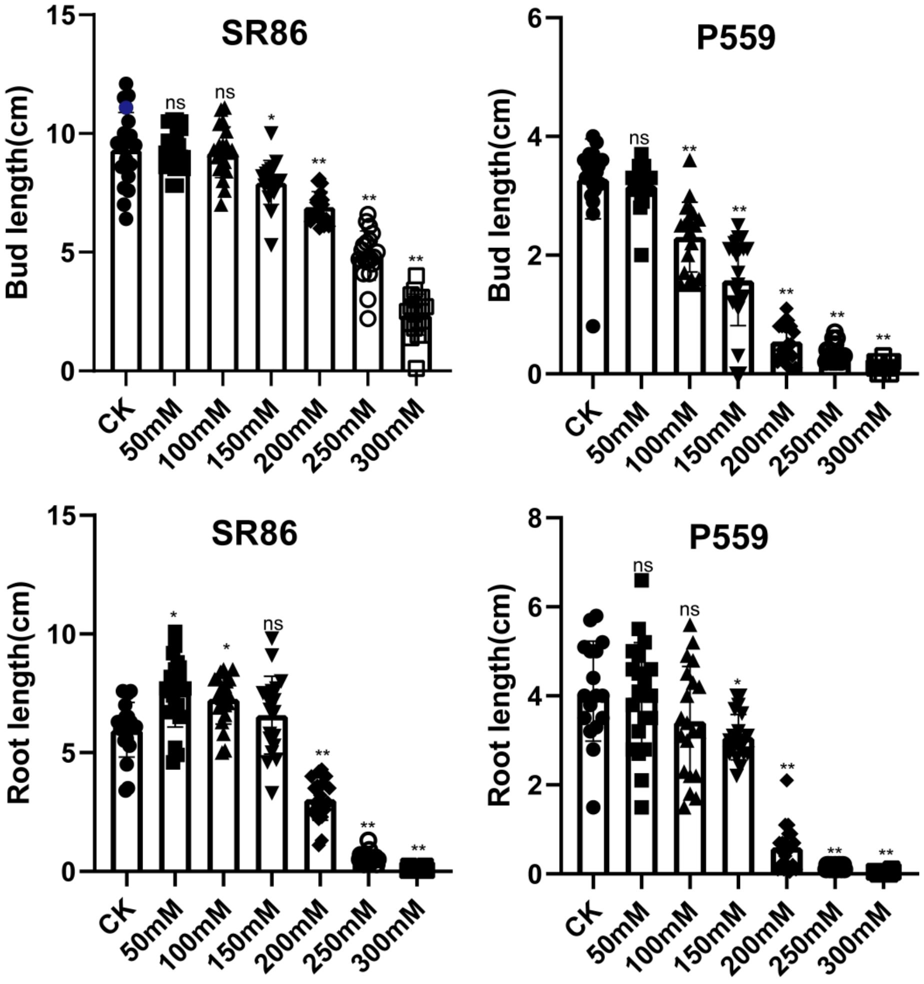

Figure S1. Effects of different salt stress concentrations on rice buds and roots after 10 days of treatment

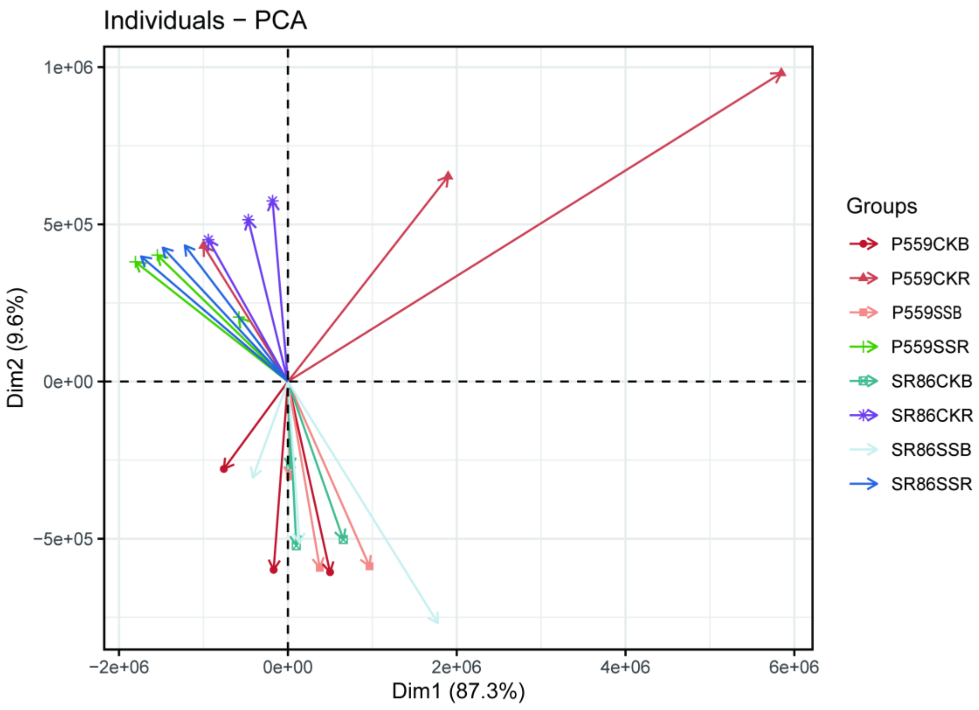

Figure S2. Principal component analysis of normalized and variance stabilized counts. Samples with similar expressions are grouped closely.

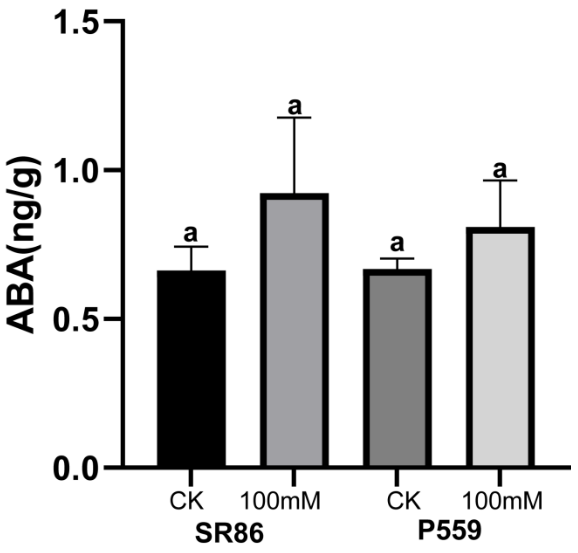

Figure S3. The content of ABA under salt stress.
